# Supplementary material for: Sizes of Long RNA Molecules Are Determined by the Branching Patterns of Their Secondary Structures
Source: Biophys J. 2016 Nov 15;111(10):2077–85. doi: 10.1016/j.bpj.2016.10.014 (PMC5113152; doi:10.1016/j.bpj.2016.10.014)
Supplement: Document S1. Figure S1 and Tables S1 and S2 [file mmc1.pdf]

**Biophysical Journal, Volume *111***

## Supplemental Information

# Sizes of Long RNA Molecules Are Determined by the Branching Patterns of Their Secondary Structures

**Alexander Borodavka, Surendra W. Singaram, Peter G. Stockley, William M. Gelbart, Avinoam Ben-Shaul, and Roman Tuma**

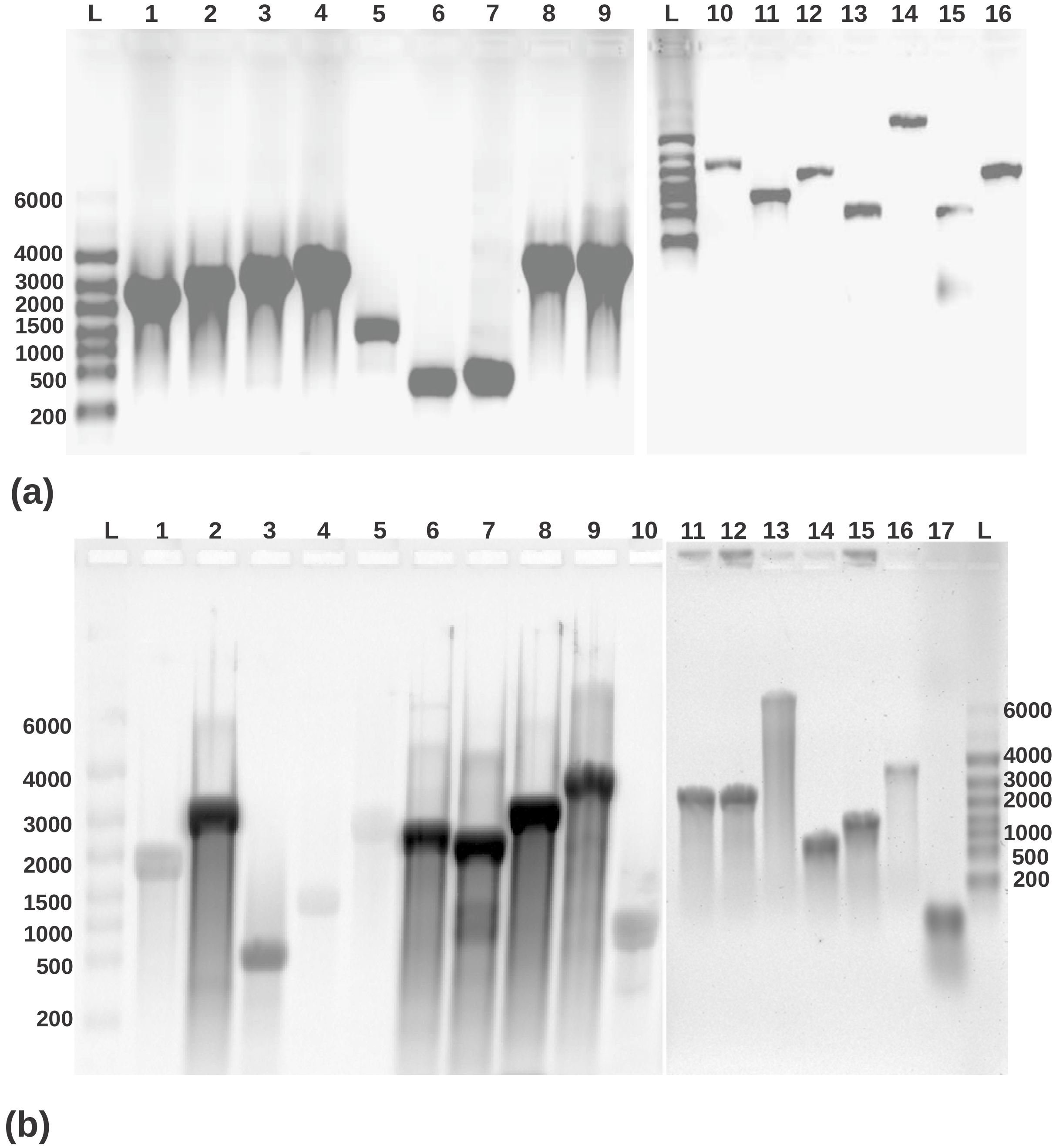


Figure S1. Denaturing formaldehyde agarose gel electrophoresis of ssRNA transcripts. (a) In vitro transcribed RNAs were separated on a formaldehyde agarose gel (1% w/v), as described below, and visualised by staining with ethidium bromide. Lane 1 – NRON; lane 2 – MS2; lane 3 – rpoB; lane 4 – MS2; lane 5 – Ef2Xlaevis; lane 6 – s11 RV; lane 7 – s11 RV after GMP-primed transcription; lane 8 – s1 RV; lane 9 – TCV; lane 10 – FHV1; lane 11 – FHV2; lane 12 – 5'RNA; lane 13 – BunV-S; lane 14 – BunV-L; lane 15 – iRNA (a 930 nt transcribed subgenomic MS2 RNA); lane 16 – 3'RNA. (b) AF488 dye-labelled RNAs visualised by fluorescence scanning upon excitation with 492 nm laser. Lane 1 – Ef2Xlaevis; lane 2 – s1 RV; lane 3 – s11 RV; lane 4 – STNV; lane 5 – 23S rRNA; lane 6 – NRON; lane 7 – HOTAIR; lane 8 – MS2; lane 9 – TCV; lane 10 – BunV-S; lane 11 – 3'RNA; lane 12 – 5'RNA; lane 13 – BunV-L, lane 14 – BunV-S; lane 15 – FHV2; lane 16 – FHV1; lane 17 – 120-mer RNA. L – RNA ladder (Thermo Scientific, #SM1821), sizes shown in number of nucleotides.

.

***Table S1: Oligonucleotide primers used for PCR cloning***

T7 promoter sequences are underlined and the first nucleotide (**G**) incorporated into RNA transcript is shown in bold.  The restriction sites utilised for linearising DNA templates are *italicised*.

| **Name of the construct** | **Primer name** | **Primer sequenceand restriction enzyme site used** |
|---|---|---|
| TCV_pSMART HC$^{Amp}$ | TCV_F1 | GTAATCTGCAAATCCCTGCACCCGCCTAAA |
| | TCV_R1 | GGGCAGGCCCCCCCCCCGC |
| | TCV_F2 | TAATACGACTCACTATA**G**GGAGAGTAATCTGCAAATCCCTGCACCCGCCTAAA |
| | TCV_R2 | ***CTCGAG***GGGCAGGCCCCCCCCCCGCG **(*Xho*I)** |
| 16SrRNA_pSMART HC$^{Amp}$ | 16S_F1 | TAAGGAGGTGATCCAACCGC |
| | 16S_R1 | AAATTGAAGAGTTTGATCATGGCTC |
| | 16S_F2 | TAATACGACTCACTATA**G**GGAAATTGAAGAGTTTGATCATGG |
| | 16S_R2 | ***TTTAAA***TAAGGAGGTGATCCAACCGC **(*Dra*I)** |
| 23SrRNA_pSMART HC$^{Amp}$ | 23S_F1 | GGTTAAGCGACTAAGCGTAC |
| | 23S_R1 | AAGGTTAAGCCTCACGGTTC |
| | 23S_F2 | TAATACGACTCACTATA**G**GGGGTTAAGCGACTAAGCGTAC |
| | 23S_R2 | ***AAGCTT***AAGGTTAAGCCTCACGGTTC **(*Hind*III)** |
| NRON_pJET1.2$^{Amp}$ | NRON_F | CACATCTCTAATGTAAACAA |
| | NRON_R | ***GATATC***TAATTACTGTTAATATCTTT **(*Eco*RV)** |
| HOTAIR_pSMART$^{Amp}$ | HotAir_F1 | CCTCCAGGCCCTGCCTTCTG |
| | HotAir_R1 | TTTATATTCACCACATGTAA |
| | HotAir_F2 | TAATACGACTCACTATA**G**GGGACTCGCCTGTGCTCTGGAG |
| | HotAir_R2 | ***GATATC***TTTTTTTTTTGAAAATGCAT **(*Eco*RV)** |

### *Table S2: DNA constructs used for RNA in vitro transcription*

| Name of DNA construct | Promoter | Restriction enzyme for linearization | Plasmid size, kb |
|---|---|---|---|
| RpoB_pSMART HC$^{Amp}$ | T7 | *Dra*I | 5.4 |
| MS2_pSMART HC$^{Amp}$ | T7 | *Hpa*I | 5.5 |
| TCV_pSMART HC$^{Amp}$ | T7 | *Xho*I | 6.3 |
| 16SrRNA_pSMART HC$^{Amp}$ | T7 | *Dra*I | 3.3 |
| 23SrRNA_pSMART HC$^{Amp}$ | T7 | *Hind*III | 4.7 |
| NRON_pJET1.2$^{Amp}$ | T7 | *Eco*RV | 5.6 |
| HOTAIR_pSMART$^{Amp}$ | T7 | *Eco*RV | 4.1 |
| *HCV JFH1/Luc SGR*$^{Amp}$ | T7 | *Xba*I | 11 |
| pUC19T7RFs1$^{Amp}$ | T7 | *Bsm*BI | 6.3 |
| pUC19T7RFs11$^{Amp}$ | T7 | *Bsm*BI | 3.6 |
| pF2100 (FHV1)$^{Amp}$ | T7 | *Xba*I | 6.2 |
| P2BS WT (FHV2)$^{Amp}$ | T3 | *Xba*I | 4.4 |
| pT7riboBUN-S$^{Amp}$ | T7 | *Bam*HI | 4 |
| BUNVL$^{Amp}$ | T7 | *Bst*UI | 10 |
| pSMART2676 (3’ MS2) HC$^{Amp}$ | T7 | *Hind*III | 4.4 |
| pSMART2578 (5’MS2) HC$^{Amp}$ | T7 | *Hind*III | 4.3 |
| pUBS-STNV-C$^{Amp}$ | T7 | *Xho*I | 3.9 |
| pTri-Xef1 (TRIPLEScript) | T7 | *Xba*I | N/A |

## RNA nucleotide sequence information

| RNA | Length, nt | Fraction of nucleotides | | | |
|---|---|---|---|---|---|
| | | A | C | G | U |
| MS2 | 3569 | 0,23 | 0,26 | 0,26 | 0,25 |
| 3' - MS2 | 2577 | 0,24 | 0,26 | 0,26 | 0,24 |
| 5' - MS2 | 2468 | 0,24 | 0,26 | 0,25 | 0,25 |
| 16S rRNA | 1542 | 0,25 | 0,23 | 0,32 | 0,2 |
| 23S rRNA | 2903 | 0,26 | 0,22 | 0,32 | 0,2 |
| BunVL | 6875 | 0,37 | 0,15 | 0,18 | 0,3 |
| BunVS | 961 | 0,31 | 0,19 | 0,22 | 0,27 |
| FHV1 | 3107 | 0,28 | 0,24 | 0,24 | 0,23 |
| FHV2 | 1400 | 0,26 | 0,26 | 0,23 | 0,25 |
| HCV | 8891 | 0,23 | 0,28 | 0,27 | 0,22 |
| HOTAIR | 2258 | 0,29 | 0,23 | 0,26 | 0,22 |
| NRON | 2730 | 0,3 | 0,24 | 0,22 | 0,24 |
| rpoB | 3555 | 0,25 | 0,26 | 0,27 | 0,22 |
| RV s11 | 667 | 0,34 | 0,2 | 0,2 | 0,27 |
| RV s11 scrambled | 667 | 0,29 | 0,23 | 0,27 | 0,2 |
| STNV | 1221 | 0,29 | 0,2 | 0,23 | 0,28 |
| TCV | 4050 | 0,27 | 0,24 | 0,27 | 0,22 |
| Ef2 | 1730 | 0,29 | 0,22 | 0,24 | 0,25 |
| RV s1 | 3302 | 0,37 | 0,16 | 0,19 | 0,28 |

## RNA sequences:

>Scrambled RNA
GCCUUUUGCAGCGCUCCGGUGACCUCCCGCGCAAGCGAGGUUUGGAGUCAUGCGUCUCUGUCGUCGACUAUAUACAA
GAGUGGAUCAUCUGCGGCCACGUCAACCCGGCUACGAUUCACUCAGGCUAGAGGGGAUCAAUAGCUCUGAGUCGAUAC
AGCACAAUUCCCUGAAUGCAAACCGUACAAGUCUCGGCGAGAAAUGAGACCAGCAGAUUGUACUUGCAACGAAUCACGG
ACCAUCUAUACGGAGAGAUCGAAAGGCGUUCAGCCUACUACCUACCAUCGCCUGUUAAUCGACUCUUAAACGAUCAAAG
GGUCAUCGGUGAACGUACGGACCUAUUGGCGGGGUUUGUCCCUUACGAAAGGAAGUAGUACGAAAGCGAACCUAGGUU
CAUGUAUACCCCUAUCAGGCAGUUCGAGUAGAGGAAAGGCCUAGGGCGACGAUAAAUCAAGGGGACUCUAGCCGGAAG
AGGAAGUGGCAUCGGAUGAAGUGGACAAGACGCUCGAGGGUGCUGAGAGUGGUGAUGAUUAACGUAAAUAUUGUAAAG
AUAAUACGAAGCCUUCCGAAAAAAGAACCAGAAUGAAACAGUGUGCAAGACAAGGGAUCGAAGAUUUGGGUCCCAAACC
UAUGAGGGCACUCGAGAGCCCGACACUCCCCUUGAGUGACG

| RNA sequence | Genbank Accession | Reference |
|---|---|---|
| 16S rRNA | AM946981.2 (region 2596124 to 2597665) | |
| HCV replicon | AB114136.1 | Targett-Adams et al. (2005) J.Gen Virol., 86:3075-80. |
| RpoB | AM946981.2 (region 4089308 to 4092862) | Borodavka et al. (2012) PNAS, 109: 39, 15769-15774. |
| 23S rRNA | AM946981.2 (region 3285031 to 3287938) | |
| FHV1 | X77156.1 | |
| NRON | NR_045006.1 | |
| MS2 | V00642.1 | Borodavka et al. (2012) PNAS, 109: 39, 15769-15774. |
| STNV | AJ000898.1 | Bringloe et al. (1998) J Gen Virol. 79:1539-46. |
| TCV | M22445.3 | Thomas et al. (2003) Virology, 306, 33–41 |
| EF-Tu X.laevis mRNA | X55324.1 | |
| BUNV-L | X14383 | Elliot RM (1989) J. Gen. Virol, 70, 1281-1285 |
| BUNVS | D00353.1 | Elliot RM (1989) J. Gen. Virol, 70, 1281-1285 |
| FHV2 | X15959.1 | Disgupta, R (1989) Nucleic Acids Res., 17 (18), 7525-7526 |
| HOTAIR | DQ926657.1 | Rinn, JL (2007) Cell 129 (7), 1311-1323 |
| RV S1 | KF729638.1 | Richards, J.E., (2013) PLoS ONE 8 (9), E74328 |
| RV S11 | KF729678 | Richards, J.E., (2013) PLoS ONE 8 (9), E74328 |
| 3’ MS2 RNA | GQ456167.1 | Rolfsson et al., J Mol Biol. 2010 Aug 13; 401(2): 309–322. |
| 5’ MS2 RNA | GQ456168.1 | Rolfsson et al., J Mol Biol. 2010 Aug 13; 401(2): 309–322. |
