# Supplementary material for: Sizes of Long RNA Molecules Are Determined by the Branching Patterns of Their Secondary Structures
Source: Biophys J. 2016 Nov 15;111(10):2077–85. doi: 10.1016/j.bpj.2016.10.014 (PMC5113152; doi:10.1016/j.bpj.2016.10.014)
Supplement: Document S2. Article plus Supporting Material [file mmc2.pdf]

Biophysical Journal
Article

Sizes of Long RNA Molecules Are Determined by the Branching Patterns of Their Secondary Structures

Alexander Borodavka,[1] Surendra W. Singaram,[2,3] Peter G. Stockley,[1] William M. Gelbart,[2] Avinoam Ben-Shaul,[3] and Roman Tuma[1,*]
[1]Faculty of Biological Sciences, Astbury Center for Structural Molecular Biology, University of Leeds, Leeds, United Kingdom; [2]Department of Chemistry and Biochemistry, University of California, Los Angeles, Los Angeles, California; and [3]The Institute of Chemistry and Fritz Haber Research Center, The Hebrew University of Jerusalem, Jerusalem, Israel

ABSTRACT Long RNA molecules are at the core of gene regulation across all kingdoms of life, while also serving as genomes in RNA viruses. Few studies have addressed the basic physical properties of long single-stranded RNAs. Long RNAs with non-repeating sequences usually adopt highly ramified secondary structures and are better described as branched polymers. To test whether a branched polymer model can estimate the overall sizes of large RNAs, we employed fluorescence correlation spectroscopy to examine the hydrodynamic radii of a broad spectrum of biologically important RNAs, ranging from viral genomes to long noncoding regulatory RNAs. The relative sizes of long RNAs measured at low ionic strength correspond well to those predicted by two theoretical approaches that treat the effective branching associated with secondary structure formation—one employing the Kramers theorem for calculating radii of gyration, and the other featuring the metric of maximum ladder distance. Upon addition of multivalent cations, most RNAs are found to be compacted as compared with their original, low ionic-strength sizes. These results suggest that sizes of long RNA molecules are determined by the branching pattern of their secondary structures. We also experimentally validate the proposed computational approaches for estimating hydrodynamic radii of single-stranded RNAs, which use generic RNA structure prediction tools and thus can be universally applied to a wide range of long RNAs.

INTRODUCTION

The discovery of ribozymes, RNA interference, and riboswitches brought RNA to the forefront of molecular biology by demonstrating that these molecules are ubiquitously involved in a wide range of cellular processes (1–4). Genome sequencing and high-throughput expression profiling have recently revealed novel long noncoding (lnc) RNAs, some of which are thousands of nucleotides long and are known to play important regulatory functions (5,6). For example, Xist lncRNA is a 17 kb-long transcript responsible for silencing one of the homologous pair of X chromosomes during mammalian development (7,8). Others, such as HOTAIR and NRON, are important regulators of gene expression (9–11) linked to diverse human diseases (12). Furthermore, a vast number of important pathogenic viruses including HIV, SARS coronavirus, poliovirus, Dengue fever virus, and many others utilize long RNAs as genetic material, which also play structural roles during virus assembly and genome packaging (13–20). Previous studies have established the importance of local secondary and three-dimensional structure in the biological function of RNA (21,22). However, the effects of the secondary structure on the large-scale properties (e.g., size) of long RNAs remain poorly understood, even while its importance for virus assembly has been demonstrated (13–20).

Several models have been developed to describe properties of double-stranded (ds) and single-stranded (ss) homopolymeric nucleic acids, both of which behave as linear polymers. Coarse-grained properties of long dsRNAs are well described by semiflexible polymer models such as the wormlike chain (23), which only take into account the overall contour length and average persistence length, the latter being weakly dependent on sequence or base composition. Similarly, the freely jointed chain model describes the conformational behavior of the more flexible single-stranded homopolymers (24,25). These models yield simple scaling laws, which relate the contour length (l) or a degree of polymerization (N, number of nucleotides) to the overall

Submitted May 26, 2016, and accepted for publication October 11, 2016.
*Correspondence: r.tuma@leeds.ac.uk
Editor: Tamar Schlick.
http://dx.doi.org/10.1016/j.bpj.2016.10.014
© 2016 Biophysical Society.
This is an open access article under the CC BY license (http://creativecommons.org/licenses/by/4.0/).
CrossMark

size, e.g., radius of gyration (R_g) or hydrodynamic radius (R_h):

$$R_g \sim R_h \sim b^{(1-\nu)} \times l^{\nu} \sim N^{\nu}. \quad (1)$$

Here ν is a scaling exponent that depends on the polymer chain model (e.g., $\nu = 0.5$ for an ideal Gaussian chain, $\nu = 0.59$ for a self-avoiding chain, and $\nu \sim 1$ for a stiff polyelectrolyte at low ionic strength), and b represents an effective segment length that is related to the persistence length (l_p) and describes polymer flexibility. Highly structured RNAs are described by a collapsed polymer chain model with ν close to 0.33, also applicable to other compact biopolymers such as globular proteins (26,27).

In contrast, due to extensive intramolecular basepairing arising from Watson-Crick complementarity of nucleotides separated by long distances along the chain contour, long ssRNAs fold into effectively branched structures with short duplex regions emanating from single-stranded loops (28) (Fig. 1). Furthermore, given the plethora of possible basepairing scenarios, thermally equilibrated long RNAs are expected to display a large number of secondary structures in solution. Notable exceptions are RNAs in large ribonucleoprotein complexes such as in ribosomes (29) or virus capsids (15,30–35). This view is supported by recent experiments confirming that protein-free viral genomic RNAs adopt an ensemble of branched conformations (28), which are further compacted upon viral assembly (14,20,36,37). Hence, selecting out a unique (native) or representative conformation is less appropriate and useful than averaging over a statistical (thermal) ensemble of secondary structures, for obtaining a reasonable estimate of the overall RNA size.

Here we examine the sizes (hydrodynamic radii, R_h) of a wide range of biologically relevant long RNA molecules at low nanomolar concentration using fluorescence correlation spectroscopy (FCS). The sizes compare well with those predicted by two ensemble averaging methods that take into account the sequence-dependent effective branching of long RNAs. Furthermore, this correlation holds even in the presence of polyvalent cations that enhance tertiary interactions and result in measurable compaction of RNAs, suggesting that these polymer theory-based methods can successfully predict sizes of long RNA molecules under a variety of conditions. Both methods are based on generic RNA structure prediction algorithms and, accordingly, would be widely applicable to other long RNAs with known sequences.

MATERIALS AND METHODS

DNA constructs used for transcribing long RNAs

MS2 phage RNA as well as the 3′ and 5′-end fragments of MS2 phage RNAs were transcribed as described in Borodavka et al. (36). The template for transcription of RpoB RNA was produced by cloning part of the open reading frame of *Escherichia coli* RNA polymerase B subunit gene (*rpoB*), as described in Borodavka et al. (14). The *Xenopus laevis* mRNA was produced by transcribing a plasmid pTRI-Xef, containing the 1.89-kbp elongation factor 1-α gene from *X. laevis* (Ambion/Thermo Fisher Scientific, Carlsbad, CA).

The TCV_pSMART_HC^{Amp} construct (Table S2 in the Supporting Material) was produced by PCR amplifying the full-length TCV cDNA using primers TCV_F1 and TCV_R1 R2 (Table S1) and a pBIN61-based vector, encompassing the full-length TCV cDNA, as a template. pBIN61-TCV plasmid was a gift from Professor George Lomonossoff (John Innes Centre, Norwich, UK). The resulting PCR product was then amplified using 5′-phosphorylated primers TCV_F2 and TCV_R2 (Table S1) to add a T7 promoter sequence to the 5′-end and an *Xho*I restriction site to the 3′-end. Further PCR product purification and cloning into pSMART HC^{Amp} vector were performed the same way as described above for the other DNA templates. Templates for transcription of 16S rRNA and 23S rRNA (16SrRNA_ pSMART_HC^{Amp} and 23SrRNA_ pSMART_HC^{Amp}) were produced by cloning the corresponding genes using genomic DNA extracted from *E. coli* BL21 cells. The primer pairs 16S_F1/16S_R1 and 23S_F1/23S_R1 (Table S1) were designed to amplify region 483879-485408 (16S ribosomal RNA, GenBank: CP001665.1) and region 228583-231490 (23S ribosomal RNA, GenBank: AM946981.2) of the BL21 DE3 *E. coli* genome. The resulting PCR products corresponding to the 16S and 23R rRNA-coding regions were used as templates for a second round of PCR amplification using 5′-phosphorylated primers 16S_F2/16S_R2 and 23S_F2/23S_R2 (Table S1), respectively. This amplification resulted in incorporation of T7 promoter sequences at the 5′-ends of both PCR products and *Dra*I (16S rRNA DNA) and *Hin*dIII (23S rRNA DNA) restriction sites at their respective 3′-ends. Further PCR product purification via agarose gel electrophoresis and subsequent cloning into a pSMART HC^{Amp} vector were performed as described above for other DNA templates. The resulting DNA constructs for in vitro transcription of 16S and 23S rRNAs are 16SrRNA_pSMART HC^{Amp} and 23SrRNA_pSMART HC^{Amp} (Table S2).

DNA template LZRS-HOTAIR (12) encompassing a 2146-nt long human HOTAIR lncRNA sequence (deposited by Professor Howard Chang, Howard Hughes Medical Institute, Stanford University, Stanford, CA) was obtained from the AddGene depository. Primers HotAir_F1 and HotAir_R1 (Table S1) were used to amplify a DNA region, corresponding to the human HOTAIR lncRNA using Q5 high-fidelity DNA polymerase (New England Biolabs, Ipswich, MA), as described above. The resulting PCR product was used as a template in a second amplification with 5′-phosphorylated primers HotAir_F2 and HotAir_R2 (Table S1), which resulted in addition of T7 promoter sequence at the 5′-end and an *Eco*RV restriction site at the 3′-end. The obtained PCR product was agarose gel-purified and used for a subsequent ligation into a pSMART HC^{Amp} vector as described above, following the manufacturer protocols. The XL1 Blue competent cells (Agilent Technologies, Santa Clara, CA) were used for transformation with the ligated products, the resulting transformants were PCR-screened, and the positive clones were verified by DNA sequencing. The resulting construct HOTAIR_pSMART HC^{Amp} (Table S2) was used for in vitro transcription of the human HOTAIR lncRNA.

cDNA for lncRNA NRON was produced by reverse-transcribing phenol-chloroform extracted total RNA from HEK 293 cells using Superscript III Reverse Transcriptase and random hexamer oligonucleotide primers (Invitrogen, Carlsbad, CA), following the manufacturer's protocol. Primers NRON_F and NRON_R (Table S1) were used to amplify the resulting cDNA using Q5 high-fidelity DNA polymerase. The resulting PCR product was agarose gel-purified and used for a subsequent ligation into a pJET1.2 vector using a CloneJET PCR Cloning Kit (Thermo Fisher Scientific, formerly Fermentas), following the manufacturer protocol. The XL1 Blue-competent cells were transformed with the resulting ligated products. The transformants were csPCR-screened and the positive plasmid clones were verified by DNA sequencing. The resulting DNA construct NRON_pJET1.2^{Amp} (Table S2) was used for in vitro transcription of the human NRON lncRNA.

Several DNA constructs for in vitro transcription were generously provided upon request by various research groups. The DNA template for

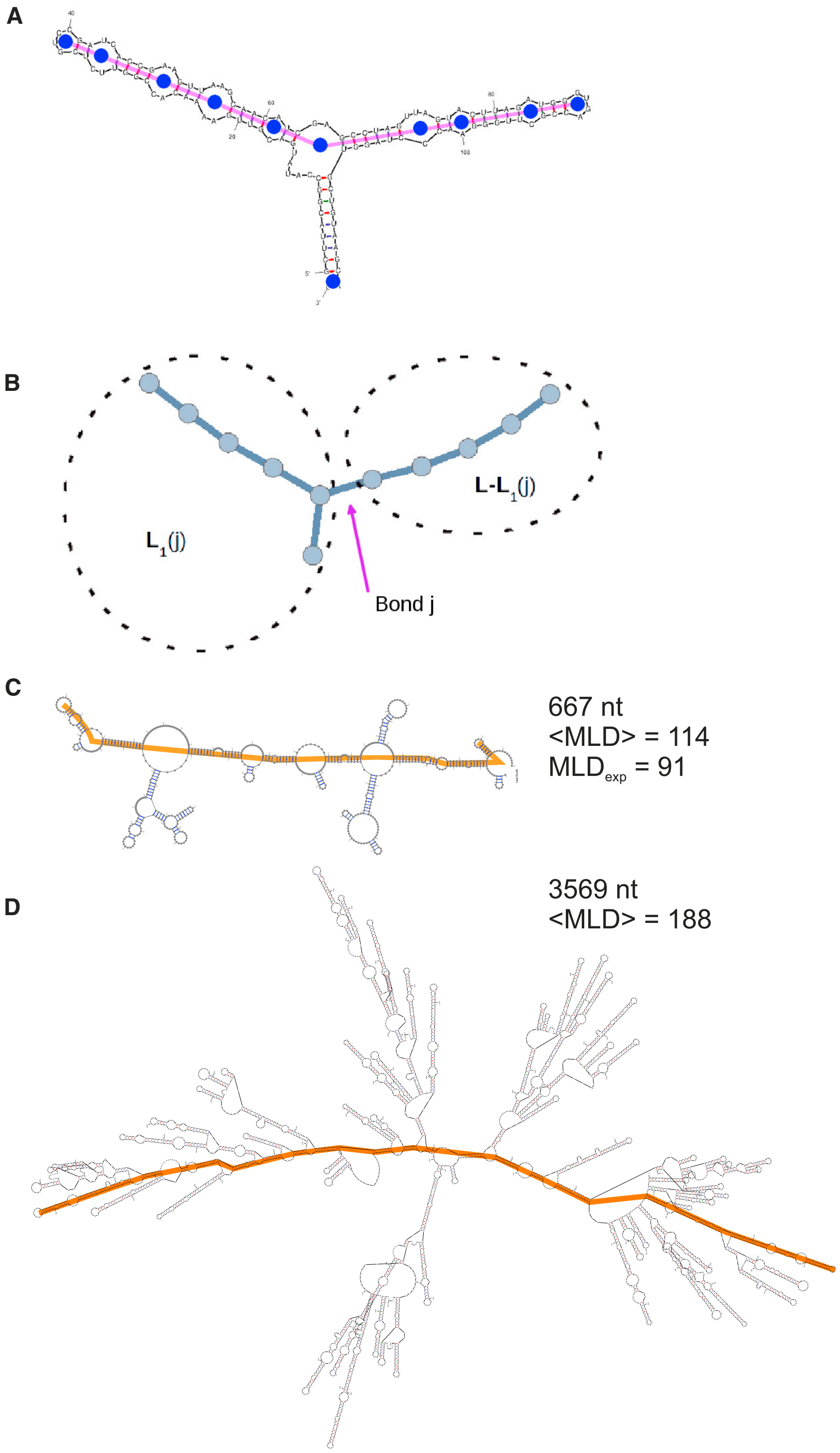

FIGURE 1 Schematics of an RNA molecule as a branched polymer. (*A*) Minimum free energy secondary structure with the maximum ladder path highlighted in magenta and flexible joints or branch points as blue dots. (*B*) Tree graph representation of the secondary structure in (*A*), with illustration of the partitioning into two halves ($L_1(j)$ and L-$L_1(j)$) at bond j for R_g computation using the Kramers theorem (see Materials and Methods). (*C*) An experimentally determined secondary structure of segment 11 (60) with maximum ladder path highlighted, and experimental $\mathrm{MLD_{exp}}$ and predicted $\langle MLD \rangle$ compared. (*D*) A representative secondary structure prediction for MS2 genomic RNA and predicted $\langle MLD \rangle$. To see this figure in color, go online.

production of the STNV-C genomic RNA was a gift from Dr. Robert Coutts (38). DNA construct HCV JFH1/Luc SGR was a gift from Professor Mark Harris (University of Leeds, Leeds, UK). DNA constructs pUC19T7RFs1 and pUC19T7RFs11 were a gift from Dr. Ulrich Desselberger (39) (University of Cambridge, Cambridge, UK). DNA constructs pF2100 and P2BS WT were donated by Professor Anette Schneemann (The Scripps Research Institute, La Jolla, CA). DNA constructs pT7riboBUN-S and BUNVL were a gift from Dr. John Barr (University of Leeds). All DNA constructs with their respective linearization restriction enzymes, used for in vitro transcription of long RNAs, are summarized in Table S2. The scrambled s11 sequence was synthesized as a gene block DNA and inserted into a pUC19 vector under control of a T7 promoter. Sequences and base compositions are summarized in the Supporting Material.

Transcription and fluorescent labeling of ssRNAs

In vitro transcription reactions were carried out using a T7 RNA transcription kit (HiScribe T7 or T3 High Yield; New England Biolabs) following the manufacturer's protocol. RNAs were purified using RNeasy mini kit (QIAGEN, Hilden, Germany) following the manufacturer's protocol, except for the fluorescently labeled RNAs. In those samples the RNA-loaded column was washed four times with 80% (v/v) ethanol before elution with 30 μL of sterile nuclease-free water. MS2-derived RNAs were 3′-end labeled while all others were 5′-end amine-modified RNAs produced by incorporation of amino-GMP and fluorescently labeled as described in Borodavka et al. (14). All RNA samples were routinely examined on denaturing formaldehyde agarose gels to ensure their integrity. Every precaution was taken to avoid contamination with RNases, and RNA samples were kept as 10 μL aliquots at $-80°C$ to minimize degradation.

FCS data collection and analysis

FCS measurements were performed on a custom-built FCS confocal setup. The excitation laser (Sapphire CW blue laser, 488 nm; Coherent, Bloomfield, CT) power was set to 65 μW. The immersion oil objective (63× magnification, numerical aperture of 1.4; Carl Zeiss, Jena, Germany) was used together with low autofluorescence immersion oil (refractive index 1.515, type DF; Cargille-Sacher Laboratories, Cedar Grove, NJ). The focus position was adjusted to 20 μm from the coverslip inner surface and precisely maintained by a piezoelectric feedback loop (Piezosystem, Jena, Germany). The photon count was recorded and analyzed by an ALV-5000 multiple tau digital correlator (www.alvgmbh.de) used in a single channel mode. Multiple runs of up to 100 autocorrelation functions with acquisition scan time of 30 s each were recorded for each of the samples using ALV-correlator software (ALV-5000/E/EPP, Ver. 3.0). Calibration of the confocal volume was performed by measuring the diffusion time of AF488-SDP dye (1 nM in RNA measurement buffer) before each data set collection. FCS data were analyzed by nonlinear least-squares fitting with a single-component diffusion model autocorrelation function corrected for the triplet state (14) using MATLAB (The MathWorks, Natick, MA). Calculation of R_h was based on the measured diffusion time value for AF488 dye and the established diffusion coefficient for a free dye using the Einstein-Stokes relationship.

RNA measurements were performed with 0.5–2 nM RNA in RNase-free 20 mM 3-(*N*-morpholino)-propanesulfonic acid (MOPS), 10 mM KOH buffer, pH 7.0 with 1 mM dithiothreitol at 25°C. RNA condensation experiments were performed in the presence of divalent (10 mM $MgCl_2$, Mg^{2+}) and trivalent (1 mM spermidine chloride, Sp^{3+}) cations, added to the 0.5–2 nM RNA samples before FCS measurements.

Theory

To account for the conformational statistics associated with an ensemble of secondary structures, it is useful (28,40–43) to represent the RNA secondary structure as a tree graph (44), i.e., a collection of points (vertices) each of which is connected by a line (bond) to at least one other point, without any closed paths. Fig. 1 illustrates this mapping for a simple case: here duplexes are treated as rigid bonds of the same length—tree edges, and single-stranded flexible loops are treated as tree vertices. Hairpin loops are vertices of order one; loops, including bulges, connecting two duplexes are twofold vertices; and loops from which three or more duplexes emanate are branched vertices (see Fig. 1 *A*). To calculate the size of the resulting branched polymer (Fig. 1 *B*), two approaches can be used. The first method makes use of the Kramers theorem (41,45,46) to directly calculate R_g from the tree topology. In the second method, the size is determined by identifying the longest chain of edges found within the tree—defined as the maximum ladder distance (MLD, Fig. 1 *A*) (42,43)—and the branched tree is replaced by a linear chain with effective contour length (N_{eff}) proportional to the MLD. Treating the resulting linear polymer as an ideal chain then gives

$$R_g = \left(b^2 N_{eff}/6\right)^{1/2}. \tag{2}$$

Here the segment length b corresponds to the average length of a duplex ($\approx$5 bp) (17,28,47) and N_{eff} is the number of duplexes along the MLD, which is $N_{eff} = MLD/b$. Thus,

$$R_g = \left(b^2 MLD/6b\right)^{1/2} \sim (MLD)^{1/2} \tag{3}$$

in bp units (42,46). The MLD is estimated from RNA secondary structure predictions and can be further refined using structure probing experiments (21). Because there is heterogeneity among the many structures whose energies lie within a thermally available range (k_BT), we use the Boltzmann-averaged MLD (denoted $\langle MLD\rangle$), derived from an ensemble of RNA structures generated by prediction algorithms implemented in RNAfold (48). Earlier theoretical analyses have shown that while even the most sophisticated and accurate basepairing programs begin to fail for long RNAs like those treated here, the relative values of their $\langle MLD\rangle$ and R_g can still be meaningfully estimated (41,42).

Size computations

Average $\langle MLD\rangle$ values were computed from the 100 lowest-energy secondary structures (42) generated using the Vienna package (48). Relative values of R_g were estimated using (see Eq. 3) the relationship $R_g \sim (\langle MLD\rangle)^{1/2}$. Each tree graph representation was derived from a dot-bracket representation of the secondary structure (see the Vienna RNA web server manual at http://rna.tbi.univie.ac.at/help.html). The R_g was calculated from the tree graph by treating the vertices as perfectly flexible joints and the edges as rigid phantom bonds (i.e., as an ideal branched polymer), and using the Kramers theorem (46). More explicitly, the R_g of a branched polymer (tree graph) was calculated by

$$\overline{R_g^2} = \left(b^2/L^2\right)\Sigma_j L_1(j)[L - L_1(j)], \tag{4}$$

where the overbar denotes an average over all conformations of the ideal branched polymer. The sum in Eq. 4 is evaluated by summing over all L bonds the product of $L_1(j)$ and L-$L_1(j)$, the numbers of vertices on either side of the jth bond (see Fig. 1 *B*). The square root of Eq. 4 yields the radius of gyration of the tree graph (i.e., $\widehat{R}_g = \sqrt{\overline{R_g^2}}$). We then averaged $\widehat{R}_g$ over the tree graphs we generated from the secondary structures, which for simplicity we refer to as the R_g (i.e., $R_g \equiv \langle\widehat{R}_g\rangle$). The predicted R_g values are reported in units of the average duplex length b.

RESULTS AND DISCUSSION

Due to their large sizes and high conformational flexibility, little is known about the structural organization and physical properties of long RNAs. Some of them, such as viral positive-sense ssRNA genomes, adopt compact conformations as part of their function and facilitate packaging into the confined space of icosahedral viral capsids (17). Likewise, several lncRNAs, including HOTAIR and SRA, assume well-defined conformations with separate domains, capable of folding into compact structures upon addition of divalent cations (49,50). These independent domains interact with their binding partners via evolutionarily conserved protein-binding motifs (49). To better understand

the architecture of long RNA molecules, e.g., their overall compactness or extendedness, we explore the relation between predicted sizes, using either the MLD or R_g obtained from Kramers theorem, respectively, and the experimentally determined hydrodynamic radii (R_h) for long RNAs, ranging from 600 to >9000 nucleotides in length.

We have examined a wide range of biologically relevant RNAs, including messenger, long noncoding, viral, and ribosomal RNAs. To minimize nonspecific intermolecular interactions between RNA molecules, we employ extremely dilute solutions (low nanomolar concentrations) and low ionic strength (i.e., good solvent conditions for charged polymers), and measure sizes of RNA molecules by FCS. In contrast to other ensemble solution techniques (small-angle x-ray and light scattering, and analytical centrifugation), the dilute conditions minimize aggregation due to intermolecular basepairing, which has previously been shown to result in an overestimation of sizes (51). Furthermore, we have also used FCS to examine compaction of individual RNA molecules in response to biologically relevant divalent (Mg^{2+}) and trivalent (spermidine, Sp^{3+}) cations. The latter conditions promote formation of tertiary structures (52,53).

Table 1 summarizes calculated $\langle MLD \rangle$ values and measured hydrodynamic radii for a range of long RNA molecules examined by FCS. Due to the low RNA concentrations and ionic strength conditions used here (notably nonphysiological, by design), aggregation and tertiary structure formation are unlikely, so that the effects of branching due to secondary structure can be accentuated and be probed directly under close-to-isolated molecule (infinite-dilution) conditions. We note that while the measured R_h broadly increases with the length, the rise significantly deviates from the monotonic behavior expected for the simple scaling laws (Eq. 1, Fig. 2 *A*). This result suggests that linear polymer scaling laws (Eq. 1) are not appropriate to describe long ssRNA, which is an effectively branched polymer. Instead, essential coarse-grained features of their sequences need to be taken into account.

To account for sequence variations, basepairing, and the resulting branching, we estimate branching patterns using the output of secondary structure algorithms (RNAfold)

TABLE 1 Hydrodynamic Radii Measured by FCS and Average Computed MLDs

Number	RNA[a]	Class[b]	Length (kb)	% BasePaired[c]	R_h Low Salt[d,e] (nm)	R_h Mg^{2+} (nm)[e,f]	R_h Sp^{3+} (nm)[e,g]	⟨MLD⟩ (rungs)[h]	R_g (a.u.)[i]
1	RV s11	ds	0.67	58	8.2 ± 1.1	11.2 ± 3.5 (9.6 ± 2)	7 ± 1.6 quenching[g]	114 ± 6	2.10
2	RV s11 scrambled	ds	0.67	56	6.5 ± 1.4	—	—	83 ± 6	2.1
3	BunVS	ss	0.96	65	10.0 ± 1.6	7.2 ± 2.1	8 ± 3.3	134 ± 11	2.23
4	STNV	ss	1.2	62	11.7 ± 1.0	8.5 ± 1.7	9 ± 2	154 ± 7	2.39
5	FHV2	ss	1.4	62	11.9 ± 2.0	9.4 ± 2.6	8.3 ± 2	176 ± 24	2.76
6	Ef2	m	1.8	60	8.8 ± 1.4	9.4 ± 1.6	9.7 ± 1.6	184 ± 14	3.12
7	16S rRNA	r	1.55	64	17.5 ± 4.0	14 ± 4.8	quenching[g]	149 ± 26	2.56
8	HOTAIR	lnc	2.4	61	16.2 ± 2.0	12.5 ± 2.4	13.4 ± 4.7	264 ± 19	3.39
9	5′-MS2	ss	2.5	69	10.7 ± 1.2	9.8 ± 0.6	10.3 ± 1.7	167 ± 17	2.74
10	3′-MS2	ss	2.6	69	13.8 ± 1.3	10.8 ± 0.8	10.5 ± 1	159 ± 9	2.68
11	NRON	lnc	2.6	58	17.6 ± 2.7	15.3 ± 3	13.7 ± 2.6	212 ± 11	3.14
12	23S rRNA	r	2.9	63	14.2 ± 2.5	11.3 ± 2.2	quenching[g]	252 ± 24	3.25
13	FHV 1	ss	3.1	62	15.6 ± 2.0	11.7 ± 4.3	9.6 ± 3.4	224 ± 13	3.12
14	RV s1	ds	3.3	58	18.4 ± 3.4	15.3 ± 2.2	18.1 ± 9 aggregation[g]	319 ± 24	3.66
15	MS2	ss	3.6	69	12.3 ± 0.6	11.3 ± 1.7	9.2 ± 1	188 ± 18	2.92
16	RpoB	m	3.6	64	18.3 ± 2.7	12 ± 1.2	10.6 ± 2	289 ± 20	3.69
17	TCV	ss	4.5	63	16.5 ± 1.7	14.7 ± 4.5	12.4 ± 4.5	341 ± 21	3.85
18	BunV L	ss	6.9	59	14.7 ± 2.4	11.7 ± 1.8	12.5 ± 2	375 ± 17	4.03
19	HCV	ss	8.9	64	33.1 ± 5.3	20.1 ± 2.6	18.8 ± 2.8	567 ± 43	4.81

[a]RV s1 and s11, human Rotavirus segment 1 and 11 precursors (single-stranded); BunVS and BunVL-Bunyamwera virus, small and large segment precursors, respectively (single-stranded); STNV, Satellite Tobacco Necrosis Virus genomic RNA; FHV1 and FHV2, Flock House Virus RNA1 and 2; Ef2 mRNA, *X. laevis* Ef2 gene transcript; 5′-MS2- 5′ end of MS2 phage genomic RNA (nucleotides 1–2469); 3′-MS2- 3′ end of MS2 phage genomic RNA (nucleotides 992–3569); TCV, Turnip Crinkle Virus genomic RNA; and HCV, Hepatitis C Virus genomic RNA.

[b]ds, single-stranded precursors of dsRNA viral genomes; ss, genomes of ssRNA viruses; m, cellular mRNA; r, ribosomal RNA; and lnc, long noncoding RNA.

[c]Percentage of basepairing averaged over 100 predictions.

[d]Measured in 20 mM MOPS-K^+, pH 7.

[e]The values are reported as average ± SD computed from at least 10 measurements. Long RNA molecules were transcribed and 5′ (or 3′; see Materials and Methods) end-labeled with Alexa Fluor 488 (Thermo Fisher Scientific), purified and subsequently checked for integrity by denaturing agarose gel electrophoresis (Fig. S1). In a few cases, quenching or aggregation affected or prevented determination of the diffusion correlation time.

[f]Measured in 10 mM $MgCl_2$ in 20 mM MOPS-K^+, pH 7.

[g]Measured in 1 mM spermidine in 20 mM MOPS-K^+, pH 7.

[h]Computed by averaging over 100 predictions (± SD).

[i]Computed using Kramers theorem.

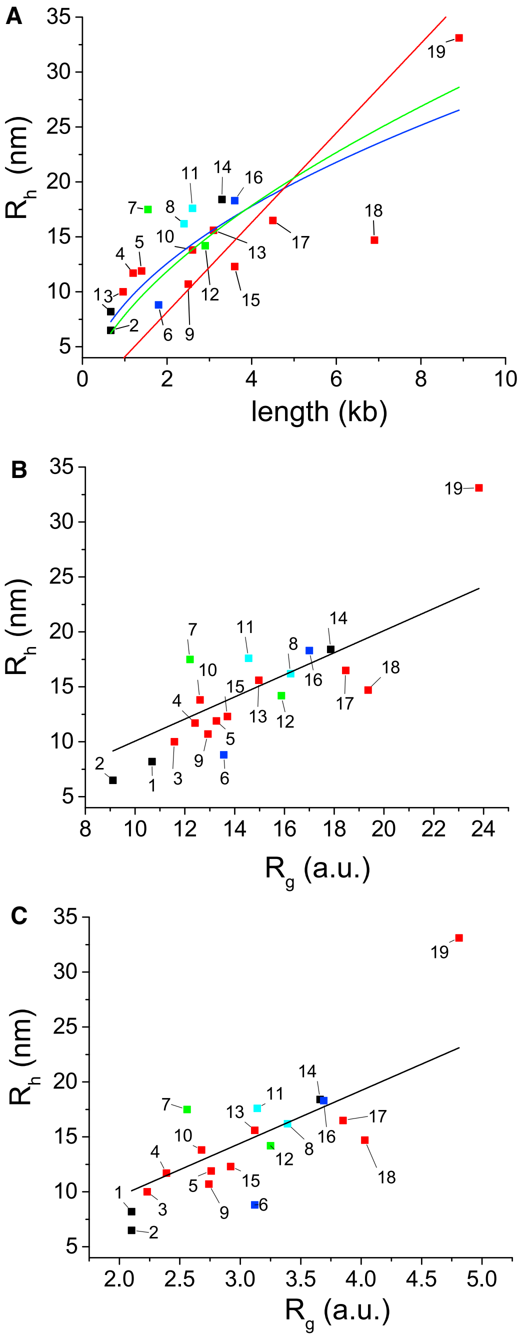

FIGURE 2 (A) Measured R_h as a function of nucleotide length (in kb). Numbering of RNAs is according to their increasing length (Table 1) and coloring is according to the class (*black*, single-stranded precursors of dsRNA viral genomes; *red*, genomes of ssRNA viruses; *blue*, cellular mRNAs; *green*, ribosomal RNA; and *cyan*, long noncoding RNAs. Lines and curves represent best fits to different linear polymer models: charged (*red*, Eq. 1, $\nu = 1$, reduced $\chi^2 = 35.85$), simple Gaussian coil (*blue*, Eq. 1, $\nu = 0.5$, reduced $\chi^2 = 13.37$), and a self-avoiding coil (*green*, Eq. 1, $\nu = 0.59$, reduced $\chi^2 = 14.85$). (*B*) Correlation between R_h and R_g predicted from $\langle MLD \rangle$ (in bp units); solid line is the best fit with reduced $\chi^2 = 11.28$. (*C*) Correlation between R_h and R_g predicted from Kramers theorem (in units of the average segment length, a.u.); solid line is the best fit with reduced $\chi^2 = 12.74$. RNA color coding and numbering is the same as in (*A*). Error bars were omitted for clarity; see Table 1 for standard deviations. To provide directly comparable reduced χ^2 values, all fitting was performed using the same nonlinear Levenberg-Marquardt algorithm in OriginPro (OriginLab, Northampton, MA). To see this figure in color, go online.

and ensemble average over the low energy structures. The measured hydrodynamic radii are in good agreement with the theoretical estimates of the R_g values based either on the MLD (Fig. 2 *B*, Eq. 3) or the Kramers theorem (Fig. 2 *C*). This result is consistent with most RNA molecules adopting branched structures in which the MLD largely determines the overall size (41,42). This is illustrated by comparing the maximum ladder path of rotavirus segment 11 precursor (s11, Fig. 1 *C*) with that of MS2 phage genomic RNA (Fig. 1 *D*). The experimentally determined secondary structure pattern of s11 is significantly less branched than that of the typical prediction for MS2. Furthermore, this is reflected in the relatively large MLD and hydrodynamic size of s11, comparable to that of Ef2 mRNA, which is three times the length. This demonstrates that the relatively simple MLD description can capture the essence of coarse-grained RNA structure, and yields quantitative predictions based on the RNA sequence alone.

Further compaction of RNA molecules and the formation of tertiary structure require di- and polyvalent cations (Mg^{2+}, spermidine^{3+}, spermine^{4+}) and/or association with RNA-binding proteins (54). As seen in Table 1, upon addition of divalent (10 mM Mg^{2+}) or trivalent cations (1 mM spermidine, Sp^{3+}), the measured R_h decreases for most RNAs, consistent with compaction driven by electrostatic screening and neutralization. Fig. 3 compares R_h before and after the addition of multivalent cations. The R_h values cluster along the line with the slope between 0.7 and 0.8, indicating that on average the RNAs undergo a 20–25% size compaction compared to their original R_h. As a consequence, the proportionality between R_h and predicted size holds for most of the RNAs even after addition of polyvalent cations. However, there are few RNAs that either fail to further compact (RV s11 No. 1 and Ef2 mRNA No. 6 in Fig. 3, where the R_h change is insignificant at confidence level 90%) or the compaction is more prominent in comparison with other RNAs examined (HCV, No. 19 in Fig. 3, where the R_h differs significantly from the expected value at confidence level 99%).

The quantitative relation between the experimental R_h and R_g predicted either from $\langle MLD \rangle$ or the Kramers theorem indicates that modeling the RNA as an ideal branched polymer constitutes a good starting point for predicting the overall size of long RNAs. However, there are several notable discrepancies between the predicted and measured sizes. One limitation of our approach is that computational predictions may yield an incorrect structure and hence an MLD that differs from that of the experimentally determined secondary structure, as in the case of STMV RNA (21,55). Such failures of the computational approach are more likely

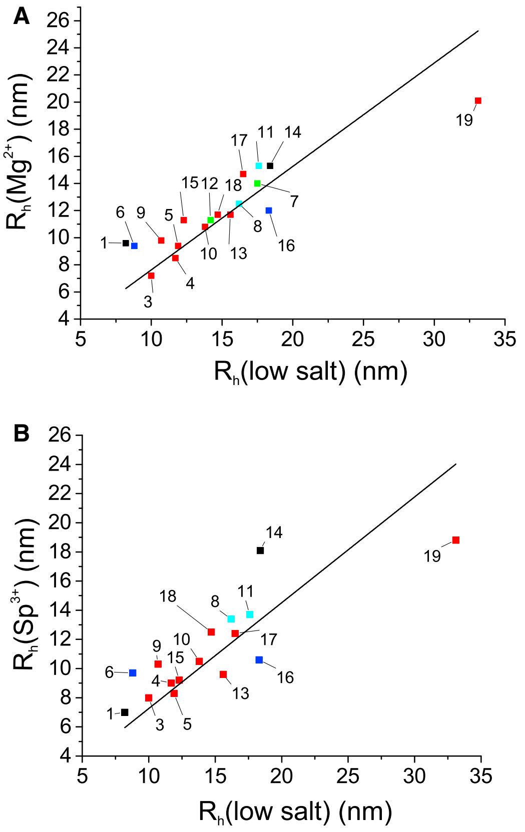

FIGURE 3 Hydrodynamic size reduction in the presence of Mg^{2+} (*A*) or spermidine Sp^{3+} (*B*). Coloring and numbering scheme is as in Fig. 2. R_h values that were compromised by either quenching or possible aggregation (RV s1 and s11 in Table 1) in the presence of multivalent cations were omitted from the plot. Linear regression lines with slopes 0.77 $\pm$ 0.03, Pearson's $r = 0.89$ for Mg^{2+} and 0.73 $\pm$ 0.04, and Pearson's $r = 0.87$ for Sp^{3+}, respectively, are shown. To see this figure in color, go online.

to occur when long RNA sequences are analyzed, thus explaining the largest deviations observed for HCV and BunV L (>5 kb), the longest RNAs examined here (Fig. 2 *B*; Table 1) (17,28). This situation can be remedied by estimating MLD from structure-probing data, which should improve the accuracy of RNA size calculations. This is demonstrated for s11 RNA for which the probing-derived MLD_{exp} is slightly lower than the computed average $\langle MLD \rangle$ (Fig. 1 *C*, compare $\langle MLD \rangle$ and MLD_{exp}), yielding a R_g ~ 9.3 value that agrees better with the experimental R_h (i.e., point No. 1 would be closer to the trend line in Fig. 2 *B*). On the other hand, when secondary structure determination (or prediction) is ambiguous, experimental size measurements by FCS can be used for selecting those structures with MLDs compatible with the experimentally determined hydrodynamic radii. In addition to the MLD prediction limitations, HCV RNA is compacted twofold in the presence of multivalent ions, i.e., to a much higher degree than other RNAs examined here (20–25% reduction), highlighting the importance of repulsive electrostatic interactions at low salt and formation of tertiary contacts stabilized by multivalent cations, not accounted for in our approach.

Another case of underestimating the size is 16S rRNA, which is predicted to be more compact than experimentally observed (Fig. 2, *B* and *C*, No. 7, R_h ~ 17.8 nm). This discrepancy likely reflects the presence of distinct domains within 16S rRNA that make the protein-free 16S rRNA relatively large (measured R_g ~11.4 nm). Only upon binding of multiple ribosomal proteins does it undergoes gradual compaction to its fully folded functional state (R_g ~ 7 nm) (56,57).

Ef2 mRNA is an example of overestimated size (Fig. 2, No. 6) and in this case tertiary contacts involving long-range interactions—not accounted for in our analysis—may play important roles in maintaining its compactness. The observed lack of further compaction of Ef2 mRNA in the presence of multivalent cations is consistent with preformed stable intramolecular contacts present in this RNA (Fig. 3, No. 6; Table 1).

To further test the observed correlation between $\langle MLD \rangle$ and the experimental hydrodynamic radius, we generated a scrambled s11 RNA sequence, as described in Materials and Methods. This disrupted as much as 25% of the original base pairings in the experimentally probed secondary structure of s11 (see sequence in the Supporting Material), while maintaining a similar level of basepairing (only 2% reduction of overall base pairing, Table 1). Analysis of the scrambled RNA sequence yields a reduction of $\langle MLD \rangle$ and is reflected in a concomitant decrease (significant at 99% confidence level) of experimentally measured R_h (Table 1; Fig. 2, RNA No. 2). In this case the native, original fold of s11 RNA is an extended conformation while the scrambled sequence produces an ensemble of more branched and hence more compact species, further demonstrating the predictive power of the MLD approach. However, the Kramers theorem approach fails to predict this reduction, most likely due to assuming the same average phantom bond length between vertices (compare to Fig. 1, *A* and *B*).

Overall, the observed differences in compactness and extendedness of RNA molecules may reflect various biological functions they perform. While MS2 phage genomic and subgenomic ssRNAs (Nos. 9, 10, and 15 in Fig. 2 *A*) are comparable in length to lncRNAs (Nos. 8 and 11) and the protein-free ribosomal RNA (No. 12), they appear to be smaller in size (see 2–4 kb region in Fig. 2 *A*). However, the Ef2 mRNA transcript (No. 6) is yet even more compact than the comparatively short viral RNAs (RNAs No. 4, 5, and 13 in Fig. 2 *A*), suggesting that although there might be evolutionary pressure on genomes of ssRNA viruses to fold into more compact structures (17), there is a number of exceptions, including more extended viral RNAs (21) and compact mRNAs. Moreover, relative size of viral RNAs may also reflect replication strategies and genome packaging mechanisms employed by viruses. For example, viruses with segmented RNA genomes may preferentially

utilize extended, less branched RNA conformations for their segment precursors (s11 in Fig. 1 *C*) to minimize the formation of nonspecific intersegment RNA-RNA contacts, while enabling formation of specific interactions facilitated by the viral RNA chaperones (58).

Remarkably, despite significant differences in the architectures of various long RNAs, we find that their sizes (hydrodynamic radii) can be estimated using coarse-grained theoretical predictions, even in the presence of multivalent ions stabilizing tertiary contacts. Because the theoretical approaches used here treat exclusively the branching patterns associated with the RNA secondary structures, our results provide experimental evidence that the overall sizes of long RNAs are determined predominantly by their secondary structure branching patterns (17). The effects of di- and polyvalent cations are more prominent for smaller RNAs, such as riboswitches and ribozymes, which adopt compact and unique tertiary structures in the presence of Mg^{2+} (59) via formation of specific tertiary contacts. Due to the heterogeneity of secondary structures in long RNAs, such specific contacts would be harder to achieve, while also explaining why long RNAs often require auxiliary proteins to guide their folding into a unique structure. This feature of RNA is likely to be the result of a limited repertoire of interactions offered by the four nucleobases and points to a fundamental limitation of RNA as a complex biopolymer when compared to proteins. We find that even relatively simple theoretical calculations based on ensembles of predicted secondary structures and MLD averaging correlate well with the experimental measurements for a diverse set of long RNA molecules, allowing our approach to account for the sizes and compactness of broad classes of ssRNAs.

SUPPORTING MATERIAL

One figure and two tables are available at http://www.biophysj.org/biophysj/supplemental/S0006-3495(16)30941-9.

AUTHOR CONTRIBUTIONS

A.B., S.W.S., P.G.S., W.M.G., A.B.-S., and R.T. designed research; A.B. and S.W.S. performed research; A.B.-S. and R.T. contributed analytic tools; and A.B., S.W.S., P.G.S., W.M.G., A.B.-S., and R.T. analyzed data and wrote the article.

ACKNOWLEDGMENTS

We thank Professor Mark Harris and Dr. John Barr (University of Leeds, UK), Dr. Ulrich Desselberger (University of Cambridge, UK), and Professor Anette Schneemann (The Scripps Research Institute, La Jolla, CA), for kindly donating plasmids JFH1/Luc SGR; pT7riboBUN-S, and pT7riboBUN-L; pUC19T7RFs1 and pUC19T7RFs11; and pF2100 and P2BS WT, which were used as templates for transcription of some of the viral RNAs.

This work was supported by the Wellcome Trust (grant Nos. 089310/09/Z and 103068/Z/13/Z to A.B.) and the Biotechnology and Biological Sciences Research Council (BBSRC) (grant No. BB/J00667X/1 to P.G.S. and R.T.).

REFERENCES

1. Zaug, A. J., and T. R. Cech. 1986. The intervening sequence RNA of Tetrahymena is an enzyme. *Science.* 231:470–475.
2. Fire, A., S. Xu, …, C. C. Mello. 1998. Potent and specific genetic interference by double-stranded RNA in *Caenorhabditis elegans*. *Nature.* 391:806–811.
3. Winkler, W., A. Nahvi, and R. R. Breaker. 2002. Thiamine derivatives bind messenger RNAs directly to regulate bacterial gene expression. *Nature.* 419:952–956.
4. Lee, R. C., R. L. Feinbaum, and V. Ambros. 1993. The *C. elegans* heterochromic gene lin-4 encodes small RNAs with antisense complementarity to lin-14. *Cell.* 75:843–854.
5. Rinn, J. L., and H. Y. Chang. 2012. Genome regulation by long noncoding RNAs. *Annu. Rev. Biochem.* 81:145–166.
6. Necsulea, A., M. Soumillon, …, H. Kaessmann. 2014. The evolution of lncRNA repertoires and expression patterns in tetrapods. *Nature.* 505:635–640.
7. Clemson, C. M., J. A. McNeil, …, J. B. Lawrence. 1996. XIST RNA paints the inactive X chromosome at interphase: evidence for a novel RNA involved in nuclear/chromosome structure. *J. Cell Biol.* 132:259–275.
8. Vallot, C., and C. Rougeulle. 2013. Long non-coding RNAs and human X-chromosome regulation: a coat for the active X chromosome. *RNA Biol.* 10:1262–1265.
9. Rinn, J. L., M. Kertesz, …, H. Y. Chang. 2007. Functional demarcation of active and silent chromatin domains in human HOX loci by noncoding RNAs. *Cell.* 129:1311–1323.
10. Willingham, A. T., A. P. Orth, …, P. G. Schultz. 2005. A strategy for probing the function of noncoding RNAs finds a repressor of NFAT. *Science.* 309:1570–1573.
11. Wapinski, O., and H. Y. Chang. 2011. Long noncoding RNAs and human disease. *Trends Cell Biol.* 21:354–361.
12. Gupta, R. A., N. Shah, …, H. Y. Chang. 2010. Long non-coding RNA HOTAIR reprograms chromatin state to promote cancer metastasis. *Nature.* 464:1071–1076.
13. Singaram, S. W., R. F. Garmann, …, A. Ben-Shaul. 2015. Role of RNA branchedness in the competition for viral capsid proteins. *J. Phys. Chem. B.* 119:13991–14002.
14. Borodavka, A., R. Tuma, and P. G. Stockley. 2012. Evidence that viral RNAs have evolved for efficient, two-stage packaging. *Proc. Natl. Acad. Sci. USA.* 109:15769–15774.
15. Dykeman, E. C., P. G. Stockley, and R. Twarock. 2014. Solving a Levinthal's paradox for virus assembly identifies a unique antiviral strategy. *Proc. Natl. Acad. Sci. USA.* 111:5361–5366.
16. Harvey, S. C., Y. Zeng, and C. E. Heitsch. 2013. The icosahedral RNA virus as a grotto: organizing the genome into stalagmites and stalactites. *J. Biol. Phys.* 39:163–172.
17. Gopal, A., D. E. Egecioglu, …, W. M. Gelbart. 2014. Viral RNAs are unusually compact. *PLoS One.* 9:e105875.
18. Comas-Garcia, M., R. F. Garmann, …, W. M. Gelbart. 2014. Characterization of viral capsid protein self-assembly around short single-stranded RNA. *J. Phys. Chem. B.* 118:7510–7519.
19. Cadena-Nava, R. D., M. Comas-Garcia, …, W. M. Gelbart. 2012. Self-assembly of viral capsid protein and RNA molecules of different sizes: requirement for a specific high protein/RNA mass ratio. *J. Virol.* 86:3318–3326.
20. Patel, N., E. C. Dykeman, …, P. G. Stockley. 2015. Revealing the density of encoded functions in a viral RNA. *Proc. Natl. Acad. Sci. USA.* 112:2227–2232.

21. Athavale, S. S., J. J. Gossett, …, S. C. Harvey. 2013. In vitro secondary structure of the genomic RNA of satellite tobacco mosaic virus. *PLoS One*. 8:e54384.
22. Ding, Y., Y. Tang, …, S. M. Assmann. 2014. In vivo genome-wide profiling of RNA secondary structure reveals novel regulatory features. *Nature*. 505:696–700.
23. Bustamante, C., J. F. Marko, …, S. Smith. 1994. Entropic elasticity of λ-phage DNA. *Science*. 265:1599–1600.
24. Seol, Y., G. M. Skinner, and K. Visscher. 2004. Elastic properties of a single-stranded charged homopolymeric ribonucleotide. *Phys. Rev. Lett.* 93:118102.
25. Sim, A. Y. L., J. Lipfert, …, S. Doniach. 2012. Salt dependence of the radius of gyration and flexibility of single-stranded DNA in solution probed by small-angle x-ray scattering. *Phys. Rev. E Stat. Nonlin. Soft Matter Phys.* 86:021901.
26. Hyeon, C., R. I. Dima, and D. Thirumalai. 2006. Size, shape, and flexibility of RNA structures. *J. Chem. Phys.* 125:194905.
27. Werner, A. 2011. Predicting translational diffusion of evolutionary conserved RNA structures by the nucleotide number. *Nucleic Acids Res.* 39:e17.
28. Gopal, A., Z. H. Zhou, …, W. M. Gelbart. 2012. Visualizing large RNA molecules in solution. *RNA*. 18:284–299.
29. Schluenzen, F., A. Tocilj, …, A. Yonath. 2000. Structure of functionally activated small ribosomal subunit at 3.3 Ångstroms resolution. *Cell*. 102:615–623.
30. Toropova, K., G. Basnak, …, N. A. Ranson. 2008. The three-dimensional structure of genomic RNA in bacteriophage MS2: implications for assembly. *J. Mol. Biol.* 375:824–836.
31. Toropova, K., P. G. Stockley, and N. A. Ranson. 2011. Visualising a viral RNA genome poised for release from its receptor complex. *J. Mol. Biol.* 408:408–419.
32. Dent, K. C., R. Thompson, …, N. A. Ranson. 2013. The asymmetric structure of an icosahedral virus bound to its receptor suggests a mechanism for genome release. *Structure*. 21:1225–1234.
33. Devkota, B., A. S. Petrov, …, S. C. Harvey. 2009. Structural and electrostatic characterization of Pariacoto virus: implications for viral assembly. *Biopolymers*. 91:530–538.
34. Johnson, J. M., D. A. Willits, …, A. Zlotnick. 2004. Interaction with capsid protein alters RNA structure and the pathway for in vitro assembly of cowpea chlorotic mottle virus. *J. Mol. Biol.* 335:455–464.
35. Johnson, K. N., L. Tang, …, L. A. Ball. 2004. Heterologous RNA encapsidated in Pariacoto virus-like particles forms a dodecahedral cage similar to genomic RNA in wild-type virions. *J. Virol.* 78:11371–11378.
36. Borodavka, A., R. Tuma, and P. G. Stockley. 2013. A two-stage mechanism of viral RNA compaction revealed by single molecule fluorescence. *RNA Biol.* 10:481–489.
37. Perlmutter, J. D., C. Qiao, and M. F. Hagan. 2013. Viral genome structures are optimal for capsid assembly. *eLife*. 2:e00632.
38. Bringloe, D. H., A. P. Gultyaev, …, R. H. A. Coutts. 1998. The nucleotide sequence of satellite tobacco necrosis virus strain C and helper-assisted replication of wild-type and mutant clones of the virus. *J. Gen. Virol.* 79:1539–1546.
39. Richards, J. E., U. Desselberger, and A. M. Lever. 2013. Experimental pathways towards developing a rotavirus reverse genetics system: synthetic full length rotavirus ssRNAs are neither infectious nor translated in permissive cells. *PLoS One*. 8:e74328.
40. Muroga, Y., Y. Sano, …, S. Shimizu. 2007. Studies on the conformation of a polyelectrolyte in solution: local conformation of cucumber green mottle mosaic virus RNA compared with tobacco mosaic virus RNA. *J. Phys. Chem. B*. 111:8619–8625.
41. Fang, L. T., W. M. Gelbart, and A. Ben-Shaul. 2011. The size of RNA as an ideal branched polymer. *J. Chem. Phys.* 135:155105.
42. Yoffe, A. M., P. Prinsen, …, A. Ben-Shaul. 2008. Predicting the sizes of large RNA molecules. *Proc. Natl. Acad. Sci. USA*. 105:16153–16158.
43. Bundschuh, R., and T. Hwa. 2002. Statistical mechanics of secondary structures formed by random RNA sequences. *Phys. Rev. E Stat. Nonlin. Soft Matter Phys.* 65:031903.
44. Zahran, M., C. Sevim Bayrak, …, T. Schlick. 2015. RAG-3D: a search tool for RNA 3D substructures. *Nucleic Acids Res.* 43:9474–9488.
45. Kramers, H. A. 1946. The behavior of macromolecules in inhomogeneous flow. *J. Chem. Phys.* 14:415–424.
46. Rubinstein, M., and R. H. Colby. 2013. Polymer Physics. Oxford University Press, New York.
47. Fang, L. T., A. M. Yoffe, …, A. Ben-Shaul. 2011. A sequential folding model predicts length-independent secondary structure properties of long ssRNA. *J. Phys. Chem. B*. 115:3193–3199.
48. Lorenz, R., S. H. Bernhart, …, I. L. Hofacker. 2011. ViennaRNA Package 2.0. *Algorithms Mol. Biol.* 6:26.
49. Somarowthu, S., M. Legiewicz, …, A. M. Pyle. 2015. HOTAIR forms an intricate and modular secondary structure. *Mol. Cell*. 58:353–361.
50. Novikova, I. V., S. P. Hennelly, and K. Y. Sanbonmatsu. 2012. Structural architecture of the human long non-coding RNA, steroid receptor RNA activator. *Nucleic Acids Res.* 40:5034–5051.
51. Strauss, J. H., Jr., and R. L. Sinsheimer. 1963. Purification and properties of bacteriophage MS2 and of its ribonucleic acid. *J. Mol. Biol.* 7:43–54.
52. Grilley, D., A. M. Soto, and D. E. Draper. 2006. Mg^{2+}-RNA interaction free energies and their relationship to the folding of RNA tertiary structures. *Proc. Natl. Acad. Sci. USA*. 103:14003–14008.
53. Draper, D. E. 2004. A guide to ions and RNA structure. *RNA*. 10:335–343.
54. Woodson, S. A. 2010. Compact intermediates in RNA folding. *Annu. Rev. Biophys.* 39:61–77.
55. Garmann, R. F., A. Gopal, …, S. C. Harvey. 2015. Visualizing the global secondary structure of a viral RNA genome with cryo-electron microscopy. *RNA*. 21:877–886.
56. Tam, M. F., J. A. Dodd, and W. E. Hill. 1981. Physical characteristics of 16 S rRNA under reconstitution conditions. *J. Biol. Chem.* 256:6430–6434.
57. Mandiyan, V., S. J. Tumminia, …, M. Boublik. 1991. Assembly of the *Escherichia coli* 30S ribosomal subunit reveals protein-dependent folding of the 16S rRNA domains. *Proc. Natl. Acad. Sci. USA*. 88:8174–8178.
58. Borodavka, A., J. Ault, …, R. Tuma. 2015. Evidence that avian reovirus σNS is an RNA chaperone: implications for genome segment assortment. *Nucleic Acids Res.* 43:7044–7057.
59. Hammann, C., and D. M. J. Lilley. 2002. Folding and activity of the hammerhead ribozyme. *ChemBioChem*. 3:690–700.
60. Li, W., E. Manktelow, …, A. M. Lever. 2010. Genomic analysis of codon, sequence and structural conservation with selective biochemical-structure mapping reveals highly conserved and dynamic structures in rotavirus RNAs with potential *cis*-acting functions. *Nucleic Acids Res.* 38:7718–7735.

Biophysical Journal, Volume *111*

Supplemental Information

Sizes of Long RNA Molecules Are Determined by the Branching Patterns of Their Secondary Structures

Alexander Borodavka, Surendra W. Singaram, Peter G. Stockley, William M. Gelbart, Avinoam Ben-Shaul, and Roman Tuma

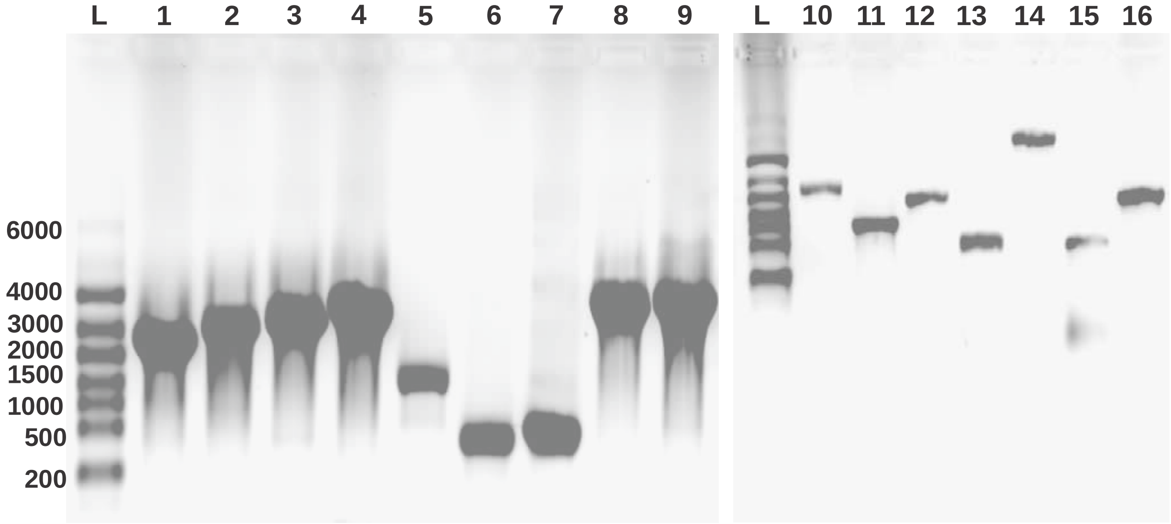

(a)

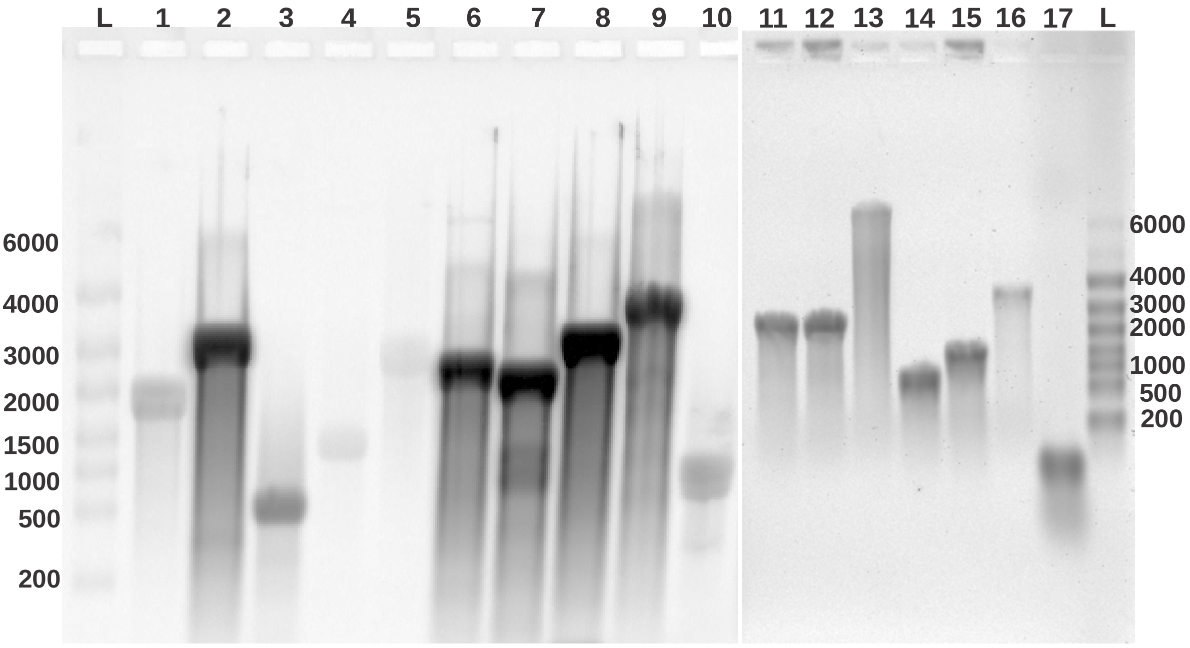

(b)

Figure S1. Denaturing formaldehyde agarose gel electrophoresis of ssRNA transcripts. (a) In vitro transcribed RNAs were separated on a formaldehyde agarose gel (1% w/v), as described below, and visualised by staining with ethidium bromide. Lane 1 – NRON; lane 2 – MS2; lane 3 – rpoB; lane 4 – MS2; lane 5 – Ef2Xlaevis; lane 6 – s11 RV; lane 7 – s11 RV after GMP-primed transcription; lane 8 – s1 RV; lane 9 – TCV; lane 10 – FHV1; lane 11 – FHV2; lane 12 – 5’RNA; lane 13 – BunV-S; lane 14 – BunV-L; lane 15 – iRNA (a 930 nt transcribed subgenomic MS2 RNA); lane 16 – 3’RNA. (b) AF488 dye-labelled RNAs visualised by fluorescence scanning upon excitation with 492 nm laser. Lane 1 – Ef2Xlaevis; lane 2 – s1 RV; lane 3 – s11 RV; lane 4 – STNV; lane 5 – 23S rRNA; lane 6 – NRON; lane 7 – HOTAIR; lane 8 – MS2; lane 9 – TCV; lane 10 – BunV-S; lane 11 – 3’RNA; lane 12 – 5’RNA; lane 13 – BunV-L, lane 14 – BunV-S; lane 15 – FHV2; lane 16 – FHV1; lane 17 – 120-mer RNA. L – RNA ladder (Thermo Scientific, #SM1821), sizes shown in number of nucleotides.

.

Table S1: Oligonucleotide primers used for PCR cloning

T7 promoter sequences are underlined and the first nucleotide (**G**) incorporated into RNA transcript is shown in bold. The restriction sites utilised for linearising DNA templates are *italicised*.

Name of the construct	Primer name	Primer sequenceand restriction enzyme site used
TCV_pSMART HC^{Amp}	TCV_F1	GTAATCTGCAAATCCCTGCACCCGCCTAAA
	TCV_R1	GGGCAGGCCCCCCCCCCGC
	TCV_F2	TAATACGACTCACTATA**G**GGAGAGTAATCTGCAAATCCCTGCACCCGCCTAAA
	TCV_R2	***CTCGAG***GGGCAGGCCCCCCCCCCGCG **(*Xho*I)**
16SrRNA_pSMART HC^{Amp}	16S_F1	TAAGGAGGTGATCCAACCGC
	16S_R1	AAATTGAAGAGTTTGATCATGGCTC
	16S_F2	TAATACGACTCACTATA**G**GGAAATTGAAGAGTTTGATCATGG
	16S_R2	***TTTAAA***TAAGGAGGTGATCCAACCGC **(*Dra*I)**
23SrRNA_pSMART HC^{Amp}	23S_F1	GGTTAAGCGACTAAGCGTAC
	23S_R1	AAGGTTAAGCCTCACGGTTC
	23S_F2	TAATACGACTCACTATA**G**GGGGTTAAGCGACTAAGCGTAC
	23S_R2	***AAGCTT***AAGGTTAAGCCTCACGGTTC **(*Hind*III)**
NRON_pJET1.2^{Amp}	NRON_F	CACATCTCTAATGTAAACAA
	NRON_R	***GATATC***TAATTACTGTTAATATCTTT **(*Eco*RV)**
HOTAIR_pSMART^{Amp}	HotAir_F1	CCTCCAGGCCCTGCCTTCTG
	HotAir_R1	TTTATATTCACCACATGTAA
	HotAir_F2	TAATACGACTCACTATA**G**GGGACTCGCCTGTGCTCTGGAG
	HotAir_R2	***GATATC***TTTTTTTTTTGAAAATGCAT **(*Eco*RV)**

Table S2: DNA constructs used for RNA in vitro transcription

Name of DNA construct	Promoter	Restriction enzyme for linearization	Plasmid size, kb
RpoB_pSMART HC^{Amp}	T7	*Dra*I	5.4
MS2_pSMART HC^{Amp}	T7	*Hpa*I	5.5
TCV_pSMART HC^{Amp}	T7	*Xho*I	6.3
16SrRNA_pSMART HC^{Amp}	T7	*Dra*I	3.3
23SrRNA_pSMART HC^{Amp}	T7	*Hind*III	4.7
NRON_pJET1.2^{Amp}	T7	*Eco*RV	5.6
HOTAIR_pSMART^{Amp}	T7	*Eco*RV	4.1
HCV JFH1/Luc SGR^{Amp}	T7	*Xba*I	11
pUC19T7RFs1^{Amp}	T7	*Bsm*BI	6.3
pUC19T7RFs11^{Amp}	T7	*Bsm*BI	3.6
pF2100 (FHV1)^{Amp}	T7	*Xba*I	6.2
P2BS WT (FHV2)^{Amp}	T3	*Xba*I	4.4
pT7riboBUN-S^{Amp}	T7	*Bam*HI	4
BUNVL^{Amp}	T7	*Bst*UI	10
pSMART2676 (3’ MS2) HC^{Amp}	T7	*Hind*III	4.4
pSMART2578 (5’MS2) HC^{Amp}	T7	*Hind*III	4.3
pUBS-STNV-C^{Amp}	T7	*Xho*I	3.9
pTri-Xef1 (TRIPLEScript)	T7	*Xba*I	N/A

RNA nucleotide sequence information

RNA	Length, nt	Fraction of nucleotides			
		A	C	G	U
MS2	3569	0,23	0,26	0,26	0,25
3' - MS2	2577	0,24	0,26	0,26	0,24
5' - MS2	2468	0,24	0,26	0,25	0,25
16S rRNA	1542	0,25	0,23	0,32	0,2
23S rRNA	2903	0,26	0,22	0,32	0,2
BunVL	6875	0,37	0,15	0,18	0,3
BunVS	961	0,31	0,19	0,22	0,27
FHV1	3107	0,28	0,24	0,24	0,23
FHV2	1400	0,26	0,26	0,23	0,25
HCV	8891	0,23	0,28	0,27	0,22
HOTAIR	2258	0,29	0,23	0,26	0,22
NRON	2730	0,3	0,24	0,22	0,24
rpoB	3555	0,25	0,26	0,27	0,22
RV s11	667	0,34	0,2	0,2	0,27
RV s11 scrambled	667	0,29	0,23	0,27	0,2
STNV	1221	0,29	0,2	0,23	0,28
TCV	4050	0,27	0,24	0,27	0,22
Ef2	1730	0,29	0,22	0,24	0,25
RV s1	3302	0,37	0,16	0,19	0,28

RNA sequences:

>Scrambled RNA
GCCUUUUGCAGCGCUCCGGUGACCUCCCGCGCAAGCGAGGUUUGGAGUCAUGCGUCUCUGUCGUCGACUAUAUACAA
GAGUGGAUCAUCUGCGGCCACGUCAACCCGGCUACGAUUCACUCAGGCUAGAGGGGAUCAAUAGCUCUGAGUCGAUAC
AGCACAAUUCCCUGAAUGCAAACCGUACAAGUCUCGGCGAGAAAUGAGACCAGCAGAUUGUACUUGCAACGAAUCACGG
ACCAUCUAUACGGAGAGAUCGAAAGGCGUUCAGCCUACUACCUACCAUCGCCUGUUAAUCGACUCUUAAACGAUCAAAG
GGUCAUCGGUGAACGUACGGACCUAUUGGCGGGGUUUGUCCCUUACGAAAGGAAGUAGUACGAAAGCGAACCUAGGUU
CAUGUAUACCCCUAUCAGGCAGUUCGAGUAGAGGAAAGGCCUAGGGCGACGAUAAAUCAAGGGGACUCUAGCCGGAAG
AGGAAGUGGCAUCGGAUGAAGUGGACAAGACGCUCGAGGGUGCUGAGAGUGGUGAUGAUUAACGUAAAUAUUGUAAAG
AUAAUACGAAGCCUUCCGAAAAAAGAACCAGAAUGAAACAGUGUGCAAGACAAGGGAUCGAAGAUUUGGGUCCCAAACC
UAUGAGGGCACUCGAGAGCCCGACACUCCCCUUGAGUGACG

RNA sequence	Genbank Accession	Reference
16S rRNA	AM946981.2 (region 2596124 to 2597665)	
HCV replicon	AB114136.1	Targett-Adams et al. (2005) J.Gen Virol., 86:3075-80.
RpoB	AM946981.2 (region 4089308 to 4092862)	Borodavka et al. (2012) PNAS, 109: 39, 15769-15774.
23S rRNA	AM946981.2 (region 3285031 to 3287938)	
FHV1	X77156.1	
NRON	NR_045006.1	
MS2	V00642.1	Borodavka et al. (2012) PNAS, 109: 39, 15769-15774.
STNV	AJ000898.1	Bringloe et al. (1998) J Gen Virol. 79:1539-46.
TCV	M22445.3	Thomas et al. (2003) Virology, 306, 33–41
EF-Tu X.laevis mRNA	X55324.1	
BUNV-L	X14383	Elliot RM (1989) J. Gen. Virol, 70, 1281-1285
BUNVS	D00353.1	Elliot RM (1989) J. Gen. Virol, 70, 1281-1285
FHV2	X15959.1	Disgupta, R (1989) Nucleic Acids Res., 17 (18), 7525-7526
HOTAIR	DQ926657.1	Rinn, JL (2007) Cell 129 (7), 1311-1323
RV S1	KF729638.1	Richards, J.E., (2013) PLoS ONE 8 (9), E74328
RV S11	KF729678	Richards, J.E., (2013) PLoS ONE 8 (9), E74328
3’ MS2 RNA	GQ456167.1	Rolfsson et al., J Mol Biol. 2010 Aug 13; 401(2): 309–322.
5’ MS2 RNA	GQ456168.1	Rolfsson et al., J Mol Biol. 2010 Aug 13; 401(2): 309–322.
